# A 20-minute, free-running, whole-heart cardiac MRI to image myocardial injury following reperfused myocardial infarction: demonstration in a large animal model

**DOI:** 10.1093/radadv/umag009

**Published:** 2026-04-17

**Authors:** Xinheng Zhang, Hsin-Jung Yang, Yuheng Huang, Ghazal Yoosefian, Keyur Vora, Khalid Youssef, Benjamin Wilk, Anthony G Christodoulou, Debiao Li, Behzad Sharif, Frank S Prato, Andreas Kumar, Rohan Dharmakumar

**Affiliations:** Department of Radiology & Imaging Sciences and Krannert Cardiovascular Research Center, Indiana University School of Medicine, Indianapolis, IN 46202-1228, United States; Biomedical Imaging Research Institute, Cedars Sinai Medical Center, Los Angeles, CA 90048, United States; Biomedical Imaging Research Institute, Cedars Sinai Medical Center, Los Angeles, CA 90048, United States; Department of Radiology & Imaging Sciences and Krannert Cardiovascular Research Center, Indiana University School of Medicine, Indianapolis, IN 46202-1228, United States; Department of Bioengineering, University of California, Los Angeles, CA 90095-1600, United States; Department of Radiology & Imaging Sciences and Krannert Cardiovascular Research Center, Indiana University School of Medicine, Indianapolis, IN 46202-1228, United States; Department of Radiology & Imaging Sciences and Krannert Cardiovascular Research Center, Indiana University School of Medicine, Indianapolis, IN 46202-1228, United States; Department of Radiology & Imaging Sciences and Krannert Cardiovascular Research Center, Indiana University School of Medicine, Indianapolis, IN 46202-1228, United States; Lawson Health Research Institute, London, ON N6A 4V2, Canada; Department of Radiological Sciences, University of California, Los Angeles, CA 90095-1600, United States; Biomedical Imaging Research Institute, Cedars Sinai Medical Center, Los Angeles, CA 90048, United States; Department of Bioengineering, University of California, Los Angeles, CA 90095-1600, United States; Department of Radiology & Imaging Sciences and Krannert Cardiovascular Research Center, Indiana University School of Medicine, Indianapolis, IN 46202-1228, United States; Department of Biomedical Engineering, Purdue University, West Lafayette, IN 47907-2032, United States; Lawson Health Research Institute, London, ON N6A 4V2, Canada; Division of Cardiology, Department of Medicine, Northern Ontario School of Medicine, Sudbury, ON P3E 2C6, Canada; Department of Radiology & Imaging Sciences and Krannert Cardiovascular Research Center, Indiana University School of Medicine, Indianapolis, IN 46202-1228, United States; Department of Biomedical Engineering, Purdue University, West Lafayette, IN 47907-2032, United States

**Keywords:** myocardial infarction, microvascular obstruction, intramyocardial hemorrhage, CCS classification of acute MI, fast cardiac MRI

## Abstract

**Background:**

Staging irreversible tissue injury in myocardial infarction (MI) enables risk assessment for post-MI major cardiovascular events. While cardiac MRI is the preferred modality for staging the severity of tissue injury, conventional scan protocols require long acquisition times with multiple breath-held and ECG-gated acquisitions, limiting its utilization.

**Purpose:**

To develop a free-breathing, whole-heart, non-ECG gated cardiac MRI for staging irreversible tissue injury in MI that can be completed in <20 minutes.

**Materials and Methods:**

A fast cardiac MRI (Biograph, Siemens Healthcare, 3 T) method based on a low-rank tensor framework was developed (reconstruction performed in MATLAB) and tested against the conventional approach using a pre-clinical canine model of reperfused MI (*n* = 15) with histological validation. Each subject underwent 2 exams that were randomized 2 days apart (day 6-8 post MI, respectively). Correlations between the proposed and conventional methods and left-ventricular ejection fraction (LVEF), MI size and transmurality, size of microvascular obstruction (MVO), and intramyocardial hemorrhage (IMH) volumes were assessed using linear regression and Bland-Altman analysis.

**Results:**

Twelve out of 15 subjects survived the initial reperfusion injury. The proposed method reduced acquisition time by >50%. The cardiac MRI evidence of tissue injury was confirmed on histopathology in all cases. The agreements between the proposed and conventional methods for LVEF, MI volume, persistent MVO volume and IMH volume were excellent; limits of agreement (LoA) were −2.1%-1.8%, −2.9%-3.3%, −2.4%-4.1%, and −1.5%-1.5%, respectively. MI transmurality and early MVO showed good agreement; LoA were −6.8%-9.7% and −6.6%-8.2%, respectively.

**Conclusion:**

The proposed free-breathing, whole-heart, non-ECG gated cardiac MRI approach permits accurate determination of tissue injury in a canine model with >2-fold reduction in scan time. While the method remains to be tested in patients, it has the potential to facilitate efficient use of cardiac MRI for staging the severity of tissue injury in patients with reperfused MI.

**Summary** 

This study validates a 20-minute, free-breathing, whole-heart, non-ECG gated cardiac MRI for staging irreversible tissue injury following reperfused myocardial infarctions in a canine model.

**Key Results** 

- A free-breathing, whole-heart, non-ECG gated cardiac MRI in a canine model can be performed within 20 minutes, reducing the scan time by half.
- Good to excellent levels of agreement were observed between the conventional and the proposed approach with respect to cardiac function, infarct size/transmurality, microvascular obstruction, and intramyocardial hemorrhage.
- The proposed approach could substantially increase scanner throughput of cardiac MRIs in patients with acute myocardial infarction.

## Introduction

Reperfusion therapy is lifesaving and has improved the immediate survival of patients experiencing ST-elevation myocardial infarction (STEMI). While myocardial infarction (MI) size is an established predictor of major adverse cardiovascular events (MACE) in this subset of patients, it has been increasingly recognized over the past decade that microvascular injury following guideline-directed reperfusion therapy is a stronger predictor of MACE compared to MI size.^1^^,^^2^ The Canadian Cardiovascular Society (CCS) classification of tissue injury defines 4 progressive stages, each associated with an increasing of MACE,^3^ with stage 1 denoting aborted MI (no tissue necrosis); stage 2 corresponding to tissue necrosis; stage 3 comprising of tissue necrosis and microvascular obstruction (MVO); and stage 4 compounding on stage 3 with intramyocardial hemorrhage (IMH).

While cardiac MRI can evaluate the various stages of tissue injury post MI, the conventional cardiac MRI protocols do not capture the complete information required for CCS staging. For instance, although IMH (CCS stage 4) is approximated to be evident in 40% of STEMI patients and is associated with worse outcomes,^4–6^ it may not be captured by the conventional cardiac MRI protocols due to multiple reasons. Firstly, the prognostic importance of IMH in STEMI patients has only evolved in the past few years. Secondly, the notion that “not all myocardial infarctions are the same” and that the severity of tissue injury is tied to post-MI MACE is only starting to be broadly recognized. Thirdly, conventional cardiac MRI acquisitions enabling complete tissue characterization can take nearly an hour, which adversely impacts scanner access and throughput. When this is combined with patient-specific requirements (multiple breath-holds, irregular cardiac rhythm, discomfort and claustrophobia) and limited spatial coverage, complete myocardial tissue characterization becomes challenging.

Cardiac MRI approaches that can permit continuous acquisition of full coverage MRI data without respiratory or cardiac gating are on the rise. Among these, MR multitasking framework^7–10^ has shown promising results, but it is not tailored for complete tissue characterization following acute MI. We developed a free-breathing, whole-heart, non-ECG gated cardiac MRI approach using low-rank tensor (LRT)-based reconstruction that can be performed in <20 minutes. The present study sought to test the proposed approach in a canine model to determine whether it can accurately identify CCS stages of irreversible tissue injury and validate it against *ex vivo* histology.

## Materials and methods

### Free-running multi-dimensional cardiac MRI with LRT reconstruction

**Figure 1:**
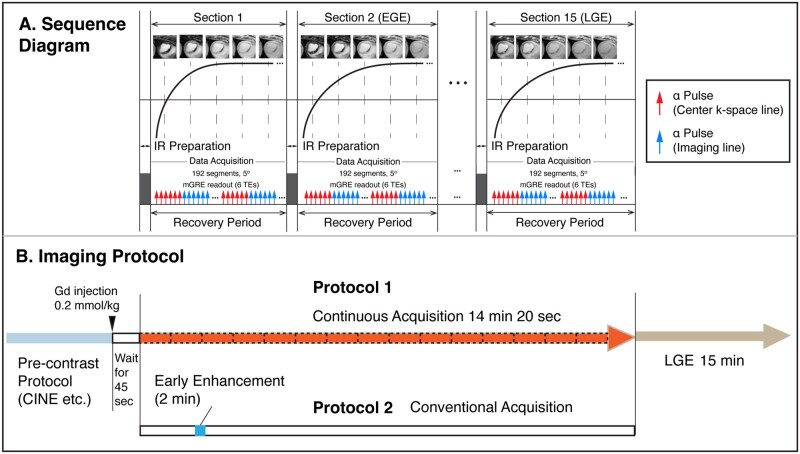
Proposed pulse sequence diagram and imaging protocol. (A). IR pulses are applied at constant intervals (2520 ms) with mGRE readouts. Following each IR preparation module, RF excitations are delivered at 5° for 192 segments. The training auxiliary data are interleaved with imaging data every 6 segments. T1 recovery and gadolinium enhancement dynamics are shown to illustrate corresponding T1 contrast changes. (B). Protocol 1: Pre-contrast acquisition (2D conventional CINE and mGRE) is followed by gadolinium contrast injection, and the sequence is run, followed by conventional late gadolinium enhancement (LGE) acquisition. Protocol 2: The same pre-contrast and LGE protocol is run but with conventional early enhancement acquisition after injection. Abbreviations: EGE, early gadolinium enhancement; Gd, gadolinium.

The LRT cardiac MRI scheme uses a custom-developed (research) sequence with a continuous randomized Gaussian-distributed 3D Cartesian k-sampling pattern to derive time-resolved T1 and T2* maps for MI characterization. The sequence details are depicted schematically in **Figure 1A**. The total acquisition duration was less than 15 minutes. The images were then reconstructed based on a previously published LRT framework,^7^^,^^9^^,^^11^ modeling the acquired data as a 6-dimensional tensor with 1 spatial dimension and 5 temporal dimensions attributable to cardiac motion, respiratory motion, temporal dynamics of gadolinium (Gd) enhancement, $T_{1}$ recovery, and $T_{2}^{*}$ decay. This allowed for fully ungated, free-breathing, 3D CINE, T1 and T2* images. The details of the image model and image reconstruction are described in **Supplementary Material S1**.

### Animal study

The proposed approach was tested in a canine model of reperfused MI (*n* = 15, body weight: 25±5 kg, age: 2-3 years). The study protocol was approved by the Institutional Animal Care and Use Committee, and the animals were studied in a whole-body 3.0 T PET/MR system (Biograph, Siemens Healthcare). Reperfused MI was created in animals using left anterolateral thoracotomy (3-hour, no-flow, ischemia of the left anterior descending coronary artery [LAD] by reperfusion).^12^ Animals surviving reperfused MI underwent 2 cardiac MRI exams in the acute phase of MI (within 6-8 days post MI) to assess tissue injury. Repeat MRI exams in each animal were performed on different days to evaluate the similarities and differences between conventional and proposed cardiac MRI approaches. Additional cardiac MRIs were performed at 8-weeks post MI to determine tissue-specific changes in the chronic phase. Prior to MRI scans, all animals were intubated and anesthetized with isoflurane (1%-1.5%/volume) with a ventilator to control their breathing.

### Cardiac MRI acquisitions

Conventional 2D breath-held ECG-gated, short-axis, pre-contrast balanced steady-state free recession CINE, multi-gradient-echo T2* images, and late gadolinium enhancement (LGE) of the whole left ventricle (LV) were acquired on either day 6 or 8 post MI. The fully ungated, whole-heart, 3D data with spatial resolution and slice location matched to the conventional 2D acquisitions were acquired 45 seconds after Gd injection (0.2 mmol/kg, **Figure 1B**) either 48 hours before or after the conventional 2D acquisition of early gadolinium enhancement (EGE). For comparison of MVO in the acute phase, conventional early Gd enhancement and late Gd enhancement were acquired 2 minutes and 15 minutes after Gd injection, respectively (**Figure 1**). Details of imaging parameters with the proposed and conventional approaches are summarized in **Table 1**.

**Table 1:** Imaging parameters.

	Proposed cardiac MRI	Conventional cardiac MRI CINE mGRE EGE/LGE		
		CINE	mGRE	EGE/LGE
**Purpose**	Cardiac function, IMH, MVO, and MI	Function	IMH	MI and MVO
**Field of view (mm^2^)**	270 × 270	270 × 185	270 × 270	270 × 270
**Matrix size**	192 × 192	192 × 132	192 × 192	192 × 192
**Resolution (mm^3^)**	1.4 × 1.4 × 6			
**Partial Fourier**	–	75%		
**GRAPPA factor**	–	R = 2		
**Inversion time (ms)**	10	–		Based on nulling time
**TE (ms)**	1.47, 3.38, 5.39, 7.4, 9.41, 11.42	1.34	1.47, 3.38, 5.39, 7.4, 9.41, 11.42, 13.43, 15.44	2.07
**Bandwidth (Hz/pixel)**	1185	930	1185	285
**TR (ms)**	13.2	2.68	17.32	4.14
**Flip angle**	5°	50°	20°	20°
**Acquisition time**	14 min 20 sec	15 sec	11 sec	17 sec
**Temporal resolution (ms)**	25.6 ± 3.8	24.6 ± 3.6	–	

Temporal resolution was retrospectively determined, thus it is dependent on heart rate. It is summarized as the mean±standard deviation (SD) across all subjects. Abbreviations: IMH, intramyocardial hemorrhage; MI, myocardial infarction; MVO, microvascular obstruction.

The scan time to acquire each 2D short-axis, breath-held, ECG-gated, cardiac CINE, T2* and LGE images was 52-57 minutes. Each, per slice, image was acquired within 1 minute, allowing for recovery from breath-holds, for a total of 12-14 slices in total (6 mm each) with full coverage of LV. For the proposed acquisition, a total of 14 partitions were acquired as part of the 3D coverage of the LV. Total acquisition time for the proposed approach was under 15 minutes. The under-sampling rate of the LRT data relative to a complete tensor was approximately 5%.

### Image reconstruction and analysis

All image reconstructions resulting from the proposed approach were performed using MATLAB 2019a (MathWorks, Natick, Massachusetts) on a Windows workstation with a 2.30-GHz dual 24-core Intel Xeon processor and 256 GB RAM, which took 9 hours per complete dataset. The reconstructed images from the proposed approach (4 respiratory and 24 cardiac phases) were subsequently converted to DICOM format and analyzed quantitatively together with conventional 2D images using cvi^42^ version 5.13.9 (Circle Cardiovascular Imaging Inc. Calgary, Canada). Regions of interest (ROIs) were delineated on cvi^42^ in consensus by 2 experienced raters (X.Z. and G.Y., > 5 years of experience). MI, MVO, and IMH were then identified using semi-automatic algorithms. To validate the accuracy of the proposed method, we measured left ventricular ejection fraction (LVEF), volume and transmurality of MI, and MVO and IMH volumes from the images of the proposed (end expiration/end-diastolic phase) and conventional methods in a blinded fashion (see **Supplementary Material S1** for additional details).

### Histological validation

Following the final imaging session, hearts were explanted and sliced along the short axis at 2-3 mm thickness. Imaging slices were matched to cardiac MRI based on fiducial landmarks (MI/IMH appearance, papillary muscles, trabecular and right ventricle insertion point geometry). It was then sectioned contiguously with 5-µm thickness and stained with Masson’s Trichrome (for fibrosis) and Prussian Blue (for iron deposits) and imaged at both 10-fold and 40-fold magnification (ECLIPSE Ni-E microscope, Nikon, Tokyo, Japan) for qualitative comparisons.

### Statistical analysis

All analyses were performed using Prism 9.2.0 (GraphPad, San Diego, California) and MATLAB. Descriptive statistics were determined for all variables as mean±standard deviation (SD). The normality of the data was assessed using the Shapiro-Wilk test, and the homogeneity of variances was evaluated using Levene’s test. Paired t-test was used to test the difference between mean MI volume, MI transmurality, MVO size/volume, and IMH volume determined using conventional and proposed approaches. Simple linear regression was performed between the measurements and regression coefficients were calculated, and the goodness of fits of the model were assessed using the R-squared values. Bland-Altman analysis was performed to determine the mean bias and limits of agreement (LoA) on 95% confidence interval (CI), and the reproducibility coefficient (RPC) was computed to test the agreement between the 2 methods. Statistical significance was set at *P* < .05.

## Results

**Figure 2:**
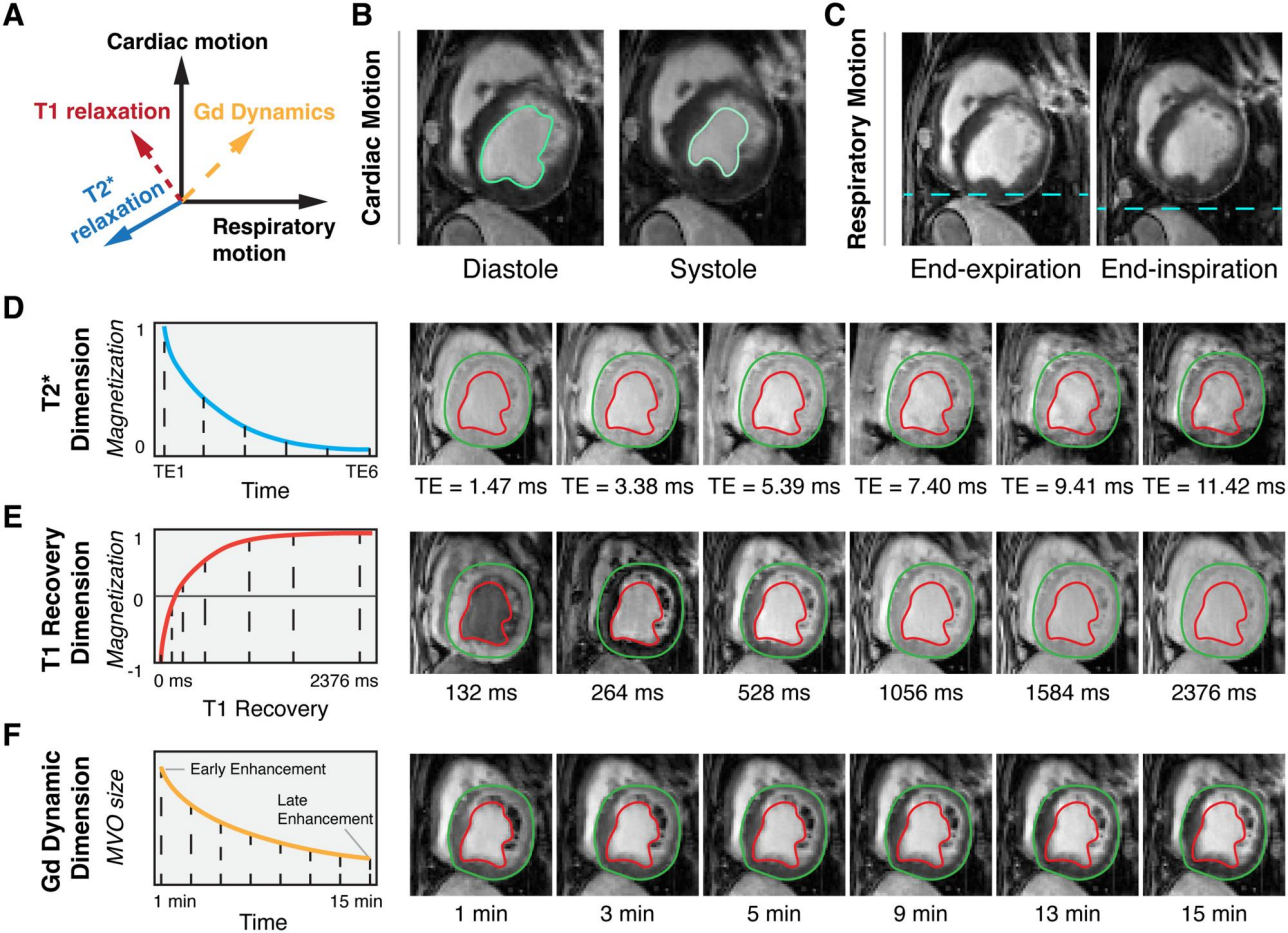
Short-axis images of a subject with myocardial infarction, microvascular obstruction, and intramyocardial hemorrhage reconstructed using the proposed approach demonstrating multiple cardiac and respiratory phases with different image contrast (T1, T2*, and gadolinium (Gd) enhancement). (A) An illustration of 2 motion dimensions (cardiac and respiratory motion) and 3 contrast dimensions (T2* relaxation, T1 recovery, and gadolinium enhancement dynamics) is shown. (B) Two cardiac phases (end-diastole and end-systole) are shown, with the blood pool highlighted by cyan color contours. (C) Two respiratory phases (end-expiration and end-inspiration), with dashed cyan color lines are drawn to highlight respiratory movement relative to the liver. (D) T2* dimension with 6 echo times (TE =1.47 to 11.42 ms) is shown; (E) T1 recovery of the myocardium following 180° inversion pulse between 132 ms to 2534 ms is shown. (F) Contrast evolution reflective of time-dependent gadolinium contrast between 1 minute and 15 minutes, fixed at 396 ms after IR pulse applied, is shown. The dashed lines on the left graphs represent corresponding MRI timepoints depicted on the right. Abbreviation: MVO, microvascular obstruction.

Twelve out of 15 subjects survived the initial reperfusion injury. The proposed method was successfully implemented as data were acquired, reconstructed, and analyzed from all animals. The images reconstructed using the proposed approach allowed for visualization of cardiac anatomy, determination of functional information, and multiple imaging contrasts (**Figure 2**). The reconstructed tensors were able to resolve cardiac motion (**Figure 2B**) and respiratory motion (**Figure 2C**) in short axis orientation with excellent image quality. **Figure 2**B-F shows representative slices along 1 short-axis view with exemplary images along the multiple temporal dimensions: cardiac motion (1 diastolic phase and 1 systolic phase), respiratory motion (1 end-expiration and 1 end-systolic), and multiple time-resolved image contrasts (T2*- and T1-weightings, along with Gd wash-out), respectively. The measures of LVEF, MI volume, MI transmurality, MVO volume, and IMH volume are summarized in **Table 2**.

**Table 2:** Measures of the proposed and conventional approach in cardiac function, myocardial infarction (MI), microvascular obstruction (MVO), and intramyocardial hemorrhage (IMH) characterization.

	Proposed	Conventional	*P* value
LVEF (%)	31.28 ± 5.42	34.34 ± 3.89	.54
MI volume (%)	16.30 ± 8.93	16.17 ± 8.23	.57
MI transmurality (%)	58.41 ± 10.07	56.97 ± 9.76	.13
MVO volume (%)	6.94 ± 5.42	6.11 ± 4.93	.17
IMH volume (%)	4.57 ± 3.30	4.57 ± 3.31	.98

The numbers are summarized as mean±standard deviation (SD) across all subjects. *P*-values were calculated from a paired t-test. Abbreviations: IMH, intramyocardial hemorrhage; LVEF, left-ventricle ejection fraction; MI, myocardial infarction; MVO, microvascular obstruction.

### Left-ventricular ejection fraction

**Figure 3:**
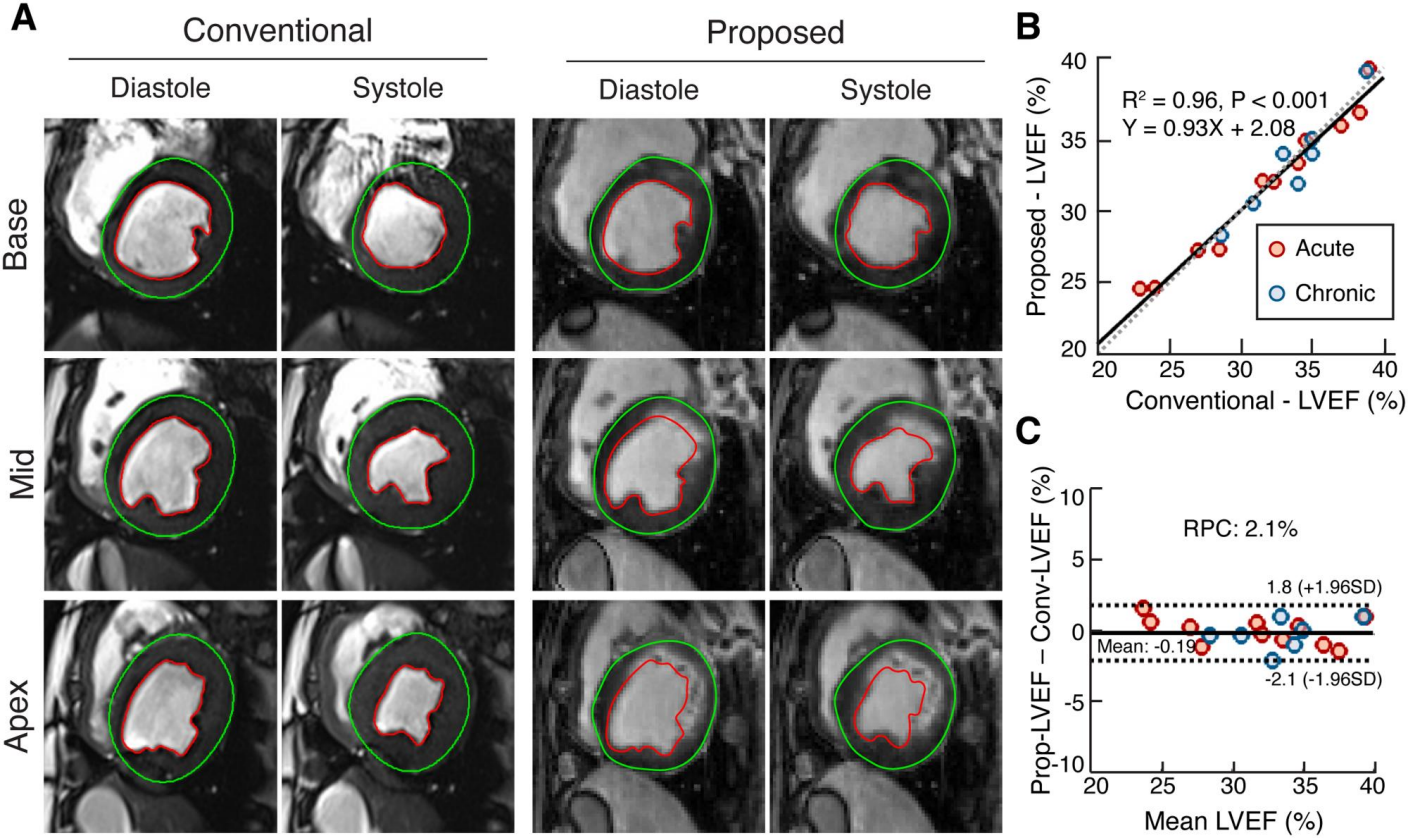
Short-axis CINE and aggregate left ventricular ejection fraction (LVEF) performance based on proposed and conventional approaches. (A) Representative end-diastolic and end-systolic short-axis images acquired at the basal, mid-ventricular, and apical sections from an animal with a reperfused left anterior descending (LAD) infarction using conventional and proposed approaches. The endocardial and epicardial contours are drawn to delineate the myocardium. (B) linear regression analysis comparing LVEF between the conventional and proposed approaches (from the aggregate data across all animals), along with the least-squares line of fit (equation, R^2^, *P* values) across the acute and chronic phases is shown. (C) Bland-Altman analyses corresponding to data in (B) along with reproducibility coefficients (RPCs) are shown.

Representative short-axis slices at end-diastolic and end-systolic cardiac phases captured during end expiration from 1 subject are shown in **Figure 3A**. The heart rates across subjects were 99.5 ± 13.7 bpm. Similar to the conventional method (2D CINE), the proposed approach allowed excellent visualization of cardiac function along with the benefit of simultaneous visualization of MI and remote regions. Linear regression analysis between the approaches showed excellent correlation (**Figure 3B**, slope = 0.93, 95% CI, [0.84, 1.03], intercept = 2.08, 95% CI, [−1.09, 5.24], R^2^ = 0.96, *P* < .001). Bland-Altman analysis also showed excellent agreement and minimal bias (mean bias was 0.19%, LoA was −2.1%-1.8%, RPC was 2.0%, **Figure 3C**).

### Infarct size

**Figure 4:**
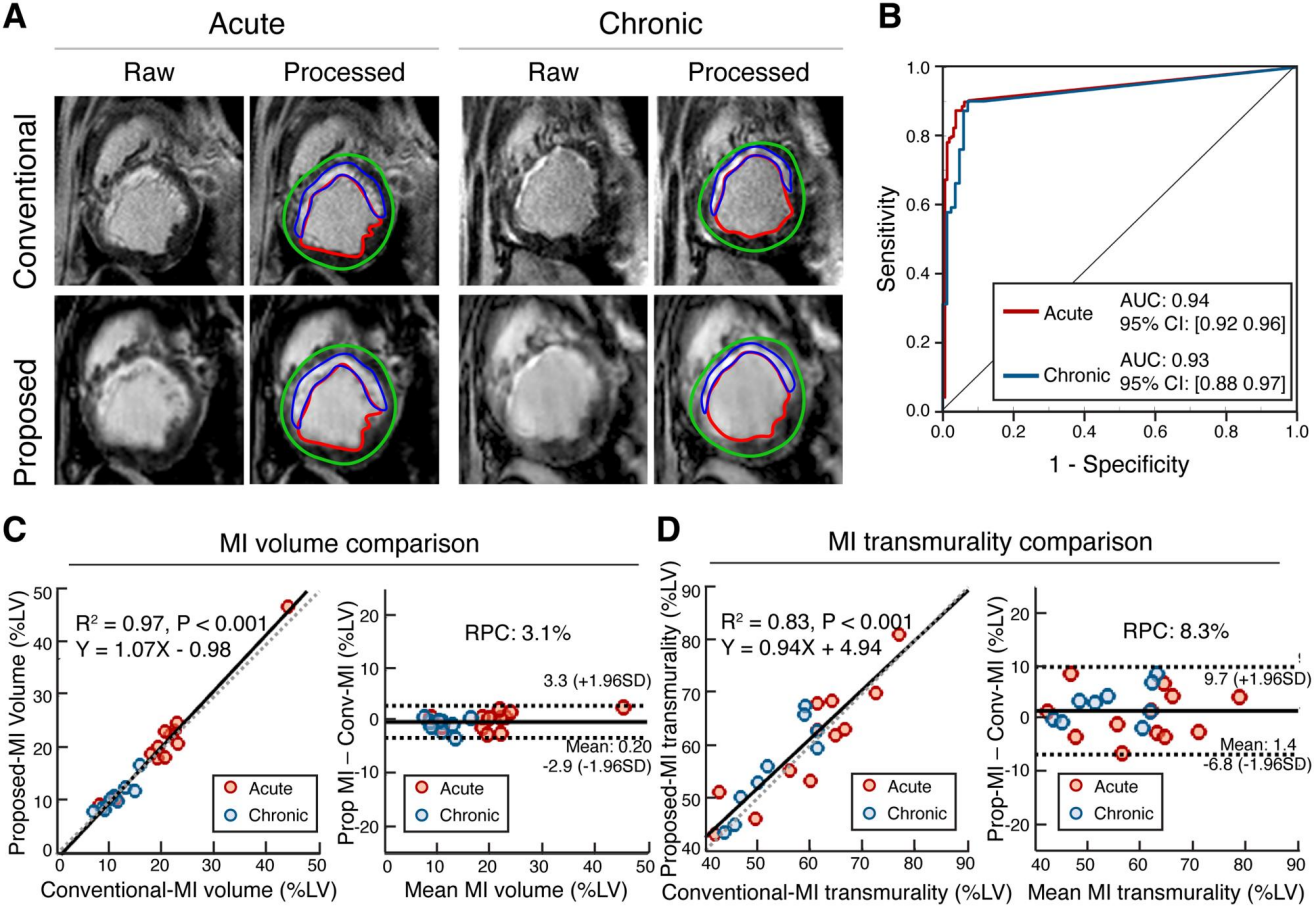
Myocardial infarct (MI) size and transmurality with proposed and conventional approaches. (A) Representative late gadolinium enhancement (LGE) images acquired using conventional and proposed approaches from an animal with a reperfused LAD infarction in the acute and chronic phases are shown. Raw images and contoured (processed) images with endocardial, epicardial and MI territory are shown. (B) ROC analyses for MI detection for subjects with acute and chronic timepoints, along with corresponding AUCs (0.94, 95% CI, [0.92, 0.96] for acute and 0.93, 95% CI, [0.88, 0.97] for chronic), are shown. (C) Linear regression analysis of MI size determined using the Conventional (Conv) and proposed (Prop) approaches, across all animals (along with line and equation of best fit, R^2^ and *P* values) and corresponding Bland-Altman analysis (along with reproducibility coefficient [RPC]) are shown. (D) Linear regression analysis of MI transmurality determined using the Conventional (Conv) and proposed (Prop) approaches, across all animals (along with line and equation of best fit, R^2^ and *P* values) and corresponding Blan-Altman analysis (along with RPC) are shown. Abbreviation: CI, confidence interval.

MI territory was clearly visible with the proposed and conventional LGE MRI (representative images are shown in **Figure 4A**). MI volume (%LV) and transmurality (%), measured from the images acquired using the proposed and conventional methods, were in strong agreement from Bland-Altman analyses: MI volume (%LV)—mean bias = 0.20%, LoA = −2.9%-3.3%, RPC = 3.1% (**Figure 4C**); and MI transmurality—mean bias = 1.4%, LoA = −6.8%-9.7%, RPC = 8.3% (**Figure 4C**), respectively, see **Figure 4**. Linear regression analysis of MI volume and MI transmurality between conventional and proposed approaches showed excellent correlation (MI volume: slope = 1.07, 95% CI, [0.99, 1.15], intercept = −0.98, 95% CI, [−2.56, 0.60], R^2^ = 0.97, *P* < .001, see **Figure 4B**; and MI transmurality: slope = 0.94, 95% CI, [0.73, 1.14], intercept = 4.94, 95% CI, [−6.94, 16.81], R^2^ = 0.83, *P* < .001, see **Figure 4D**). ROC analysis showed that the AUC for detection of acute MI was 0.94 with 95% CI, 0.92 to 0.96; and chronic MI was 0.93 with 95% CI, 0.88 to 0.97, see **Figure 4B**).

### MVO: early enhancement and persistent (late) MVO

**Figure 5:**
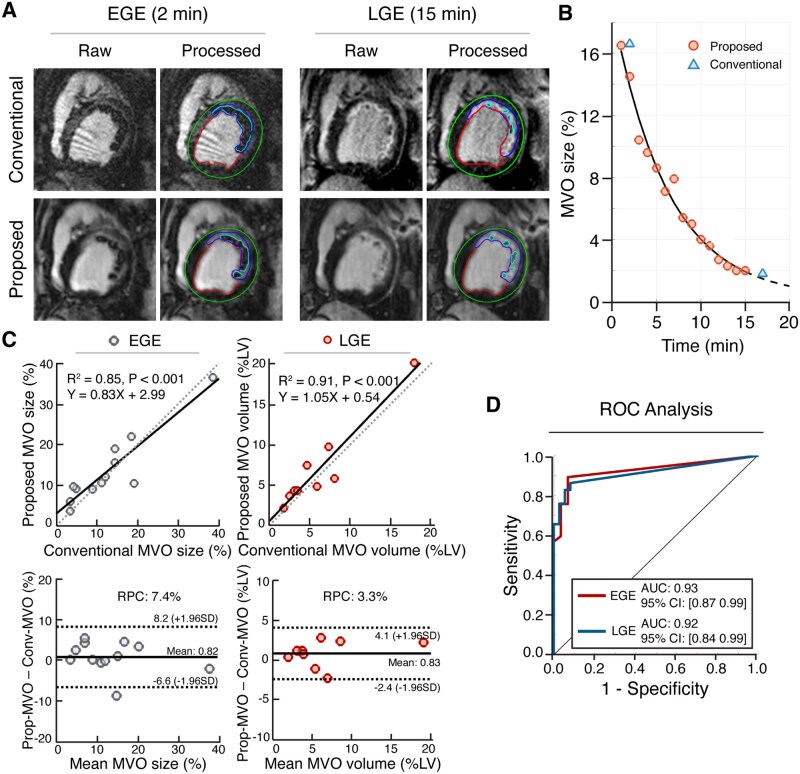
Time-dependent (early and persistent) microvascular obstruction (MVO) characterization using proposed and conventional approaches. (A) Representative early gadolinium enhancement (EGE) and late gadolinium enhancement (LGE) images acquired using the proposed and conventional methods are shown. Processed images delineate the endocardium (red), epicardium (green), MI region (blue), and MVO zone (cyan). (B) Representative time-dependent MVO sizing acquired using the proposed and conventional approaches for the animal in (A) are shown. (C) Linear regression plots of early MVO (from EGE) and persistent MVO (from LGE) and corresponding Bland-Altman plots from aggregate data are shown with (line of best fit and corresponding equation, R^2^, P; and reproducibility coefficients [RPCs]). (D) ROC analyses between the 2 methods for identifying early and persistent MVO based on EGE and LGE are shown along with corresponding AUC (0.93, 95% CI, [0.87, 0.99] for acute; and 0.92, 95% CI, [0.84, 0.99] for chronic). Abbreviation: LV, left ventricle.

To validate early MVO and persistent MVO (determined from late enhancement, PMO), comparison of early enhancement (2 minutes post Gd, EGE) was made with conventional and proposed approaches in the acute phase of MI (see **Figure 1B**). MVO was not assessed at chronic phase, as consistent with the literature,^13^ there was no evidence of MVO in the chronic phase. The single-slice 2D conventional EGE was compared to the proposed approach (after matching for slice positions), while persistent MVO (%LV) was compared using corresponding whole-heart LGE. Representative EGE and LGE images acquired from the same subject are shown in **Figure 5A**. Dynamic changes in MVO as a function of time after Gd injection, measured using both the conventional and proposed approaches, are shown in **Figure 5B**. Good agreement was found between the 2 methods for EGE and persistent MVO. MVO volume (%LV) determined using proposed vs conventional: mean bias was 0.82%, LoA was −6.6%-8.2%, RPC was 7.4% for EGE, mean bias was 0.83%, LoA was −2.4%-4.1%, and RPC was 3.3% for persistent MVO. Linear regression of EGE and persistent MVO from the conventional and proposed approaches were strongly correlated (**Figure 5C**, slope = 0.83, 95% CI, [0.58, 1.07], intercept = 2.99, 95% CI, [−0.87, 6.85], R^2^ = 0.85, *P* < .001 for EGE; slope = 1.05, 95% CI, [0.75, 1.35], intercept = 0.54, 95% CI, [−1.75, 2.83], R^2^ = 0.91, *P* < .001 for persistent MVO). There was a strong correlation and agreement in MVO quantification with the conventional and proposed approaches. ROC analysis showed that the AUC for MVO detection with EGE and persistent MVO were 0.93 (95% CI, 0.87 to 0.99) and 0.92 (95% CI, 0.84 to 0.99), respectively (**Figure 5D**).

### Intramyocardial hemorrhage

**Figure 6:**
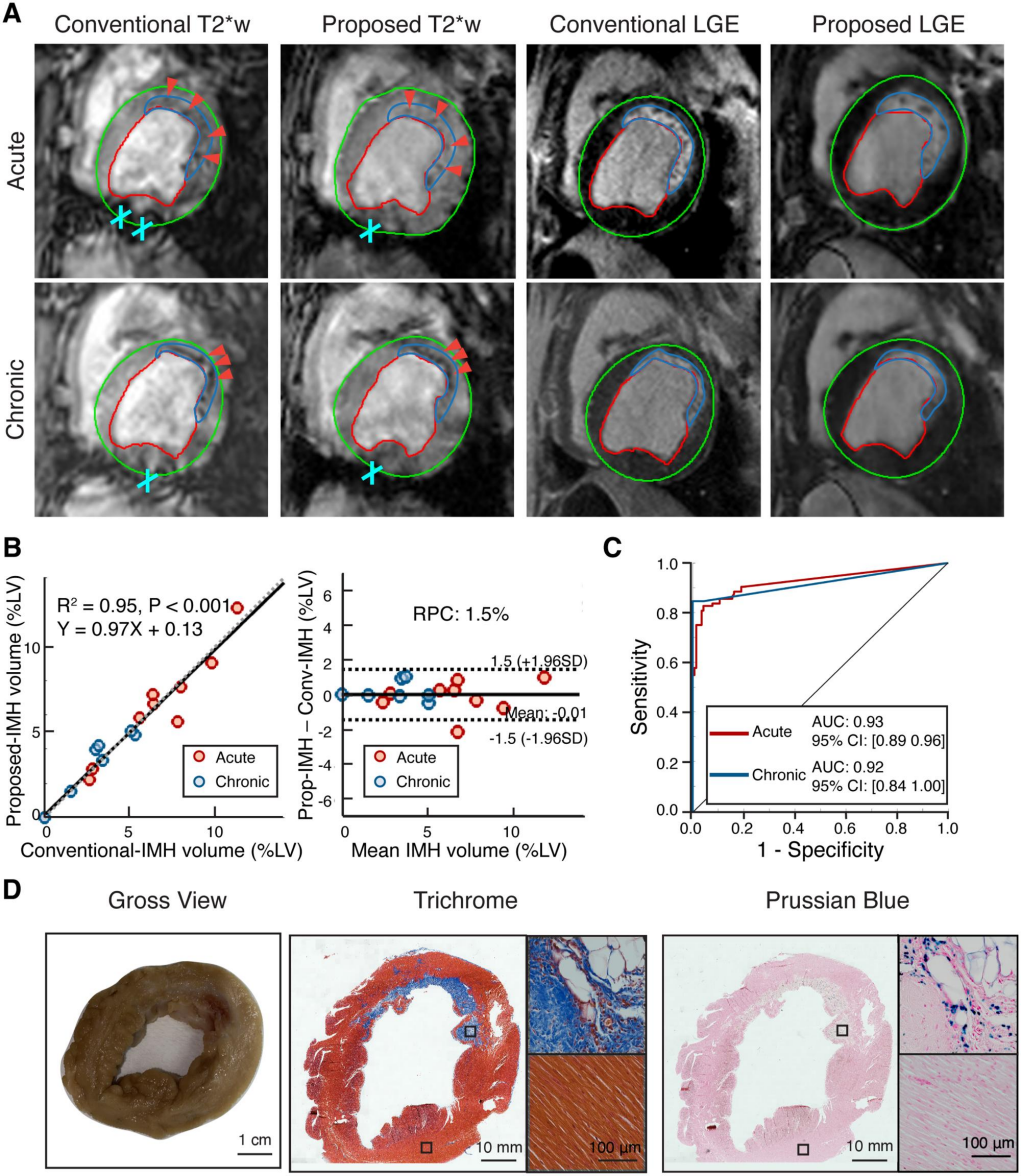
Determination of intramyocardial hemorrhage (IMH) with proposed and conventional approaches along with histological validation of hemorrhagic remnant (iron) and focal fibrotic tissue (chronic myocardial infarction [MI] regions). (A) Representative T2*-weighted (TE = 11.43 ms) and late gadolinium enhancement (LGE) images acquired using conventional and proposed approaches from an animal with reperfused LAD infarction in the acute and chronic phases are shown. Delineation of the epicardium, endocardium and MI region are shown. IMH is identified with arrowheads, and off-resonance artifacts are identified with cross. (B) Linear regression analysis of IMH size (%LV) determined using the Conventional (Conv) and proposed (Prop) approaches, across all animals across both acute and chronic phases (along with line and equation of best fit, R^2^ and *P* values) and corresponding Bland-Altman analysis (along with reproducibility coefficient [RPC]) are shown. (C) ROC analyses of IMH detection across acute and chronic phases are shown along with corresponding AUCs (0.93, 95% CI, [0.89, 0.96] for acute and 0.92, 95% CI, [0.84, 1.00] for chronic). (D) Short axis cross-sections of the heart (gross view, along with Trichrome and Prussian blue contiguous sections corresponding to the MR image) are shown. Masson’s Trichrome and Prussian Blue are shown at 10-fold magnification of images stitched together for a whole slice view, along with MI and remote zones magnified with 40-fold magnification. Scale bars of the gross optical image, histological image, and 40-fold magnified histological image are overlaid on the bottom left. Abbreviation: LV, left ventricle.

IMH volume (%LV) was measured from T2*-weighted images reconstructed using the proposed and conventional approaches. Representative T2* weighted and LGE images obtained using the proposed and conventional approaches, along with corresponding histological sections obtained after the chronic imaging study are shown in **Figure 6**. Visually both approaches provided good correspondence in size and location of IMH within the MI zone as identified by LGE images (see **Figure 6A**). Notably, the proposed approach showed greater resistance to off-resonance artifact than the conventional approach.

IMH size determined using the proposed and conventional approaches were in excellent agreement: mean bias = −0.01%, LoA = −1.5%-1.5%, RPC = 1.5% (**Figure 6B**). Linear regression analysis of IMH size between the conventional and proposed approaches showed excellent correlation (slope = 0.97, 95% CI, [0.85, 1.09], intercept = 0.13, 95% CI, [−0.53, 0.79], R^2^ =0.95, *P* < .001; see **Figure 6B**). ROC analysis of IMH detection using the proposed approach showed that the AUC for acute and chronic phases were: 0.93 (95% CI, 0.89 to 0.96) and 0.92 (95% CI, 0.84 to 1.00), respectively (see **Figure 6C**). Consistent with T2* data, Elastin-Mason Trichrome stain (EMT) and Prussian Blue stain showed colocalization of iron within MI (see **Figure 6D**). **Supplementary Material S2** provides additional details on MI characteristics based on the proposed approach in the acute and chronic phases (see **Supplementary Material S2**).

## Discussion

We developed a free-breathing, non-ECG-gated acquisition scheme allowing for a comprehensive qualitative and volumetric characterization of tissue injury in reperfused MI with >50% reduction in the total acquisition time. We demonstrated that the proposed approach yields good to excellent agreement of LVEF, MI volume and transmurality, MVO, and IMH volume compared to the conventional 2D approach with whole-heart coverage. We demonstrated at both acute (6-8 days after MI) and chronic (8 weeks) MI phases of MI using a large animal model using an imaging protocol that could be performed within 20 minutes. While the authors recognize that not all MI characterization protocols currently employed require an hour-long scan,^14^ those that would yield comprehensive characterization of MI (inclusive of IMH) with volumetric information enabling all forms of tissue injury allowing for accurate CCS staging will necessitate 60 minutes of scan time. Thus, the proposed approach yielding over 50% reduction in scan time is a significant advance.

Standard MRI protocols allowing for whole-heart coverage with multiple breath holds can take more than an hour (assuming 14 slices, each slice taking 1 minute including rest time between breath-holds; cardiac localizers [3-5 minutes]; CINE for cardiac function [14 minutes], T2* MRI for IMH detection [14 minutes], 10 to 15 minutes delay/wait time after contrast injection for LGE [14 minutes]). Depending on the purpose, CINE acquisition could be performed after the contrast injection, which can reduce the scan time to under 48 minutes. The proposed 3D method provides multiple advantages. It reconstructs multiple co-registered contrasts from a single 14.3-minute free-running acquisition, which when combined with a localization protocol (3-5 minutes) can be completed within 20 minutes. This framework uses an inversion-recovery module that enables retrospective selection of the optimal inversion time for MI visualization. The continuous 2D Gaussian sampling eliminates ECG triggering, breath-holds, and navigators, improving efficiency and enabling phase-resolved cine reconstruction with reduced off-resonance and motion artifacts. Continuous scanning also overcomes the limited temporal window of early MVO imaging by capturing dynamic contrast changes throughout the entire acquisition. Finally, LGE volumes acquired at 14-15 minutes can match conventional 2D LGE, confirming consistency across MI size.

We found that both MI transmurality and early MVO characterization showed slight deviations (RPC > 5%) between the proposed and conventional approaches. For MI transmurality, the deviation reflects the inherent challenges associated with defining anatomical RV insertion points, and potential differences in the motion-resolved cardiac phase used for analysis. Both factors are known to influence transmurality estimation and may contribute to the observed variation. For early MVO, it was challenging to validate the early enhancement images acquired using the 2 methods due to (a) slice mismatch between the acquisitions; and (b) the conventional 2D acquisitions were acquired within 10-15 seconds of contrast injection while the proposed approach took nearly a minute, potentially resulting in differences in contrast clearance during the acquisitions. These discrepancies could explain the slight deviations in the appearance of early MVO as seen in **Figure 5B**.

Besides prognostication for MACE, interventions to mitigate tissue damage at the various CCS stages of injury are evolving,^15–17^ and an assessment of their effectiveness would require a cardiac MRI evaluation. While an important step was taken by the Society for Cardiac Magnetic Resonance^14^ in promoting a 30-minute protocol for tissue characterization, it (1) misses IMH characterization; (2) demands frequent patient breath-holds, potentially increasing the risk for motion artifacts; and (3) relies on experienced technologists to quickly navigate and adjust the scans. The proposed cardiac MRI approach overcomes these limitations through continuous data acquisition without respiratory or ECG gating with 3D volumetric coverage.

The proposed framework was implemented and tested using a canine model in a 3 T MRI system. For clinical use, additional considerations may be required. (1) Patients may require a larger field-of-view (FOV) to cover the whole torso. This means the acquired matrix size will be larger (assuming spatial resolution is the same), resulting in a lower sampling rate. This issue can be mitigated with techniques employing variable or non-cartesian 3D trajectories to increase scan efficiency.^9^ (2) Patients may exhibit greater irregular motions (eg, arrhythmia, breathing pattern, etc.). Although the current framework incorporates self-navigation for retrospective cardiac and respiratory gating, motion variability may introduce residual motion artifacts. In such cases, integrating advanced respiratory motion models such as nonrigid neural-network-based methods^18^^,^^19^ for motion compensation could further improve the robustness of the proposed approach in these patient populations. (3) Patients with implanted cardiac devices (eg, pacemakers, ICDs etc.) may pose challenges due to susceptibility-induced signal loss and B0 inhomogeneity, which may be overcome with advanced dynamic shimming to minimize the field inhomogeneity.^20^

### Study limitations

The following limitations need to be acknowledged. Firstly, the IMH characteristics between conventional and proposed methods were compared at different stages of contrast administration (conventional T2* images were collected pre-contrast and T2* images were collected following contrast administration). Nonetheless, consistent with other pilot studies,^21^ no difference in IMH volume was observed between the testing conditions using the 2 methods. Secondly, while the present approach allows for characterization of CCS stages II, III, and IV, its capability for identifying CCS stage I was not assessed. This would have required an inclusion of another animal model where ischemia followed by reperfusion results in myocardial edema (reversible injury) in the absence of MI. It would also have required the incorporation of T2 imaging to discern myocardial edema. Thirdly, the reconstructions are performed off-line and at a high computational cost (∼9 hours), which stems from iterative optimization procedures in manipulating multi-dimensional tensors. Reconstruction time could be shortened by GPU implementation, optimizing the framework (eg, randomized SVD^22^), or data-consistent deep learning approaches^23^ and may be implemented online using an open-source reconstruction framework such as Gadgetron.^24^^,^^25^ Finally, spatial blurring is a known limitation of the proposed LRT reconstruction, which results from the global low-rank constraints that are imposed to balance between blurriness and noise reduction resulting in over-smoothening of fine structural details (see **Supplementary Material S3**). This limitation may be overcome by incorporating advanced edge-preserving regularization^26^ or local processing in low-rank approximation.^27^

## Conclusion

We have developed a cardiac MRI approach and tested it using a large animal model, which allows for CCS classification of key stages of irreversible tissue injury in reperfused MI without the need for breath holding and ECG-gating which can be completed in less than half the time as standard protocols. The proposed approach can allow for characterization of cardiac function, infarct size and transmurality, time-resolved assessment of MVO, and IMH. Although the proposed method remains to be tested in patients, it is expected to facilitate the efficient use of cardiac MRI for CCS staging of revascularized MI patients.

## Supplementary Material

umag009_Supplementary_Data

## Data Availability

The data underlying this article will be shared on reasonable request to the corresponding author.

## Abbreviations

EGE: early gadolinium enhancement
IMH: intramyocardial hemorrhage
LGE: late gadolinium enhancement
LRT: low-rank tensor
LV: left ventricle
LVEF: left-ventricular ejection fraction
MACE: major adverse cardiovascular events
MVO: microvascular obstruction
RPC: reproducibility coefficient
STEMI: ST-elevation myocardial infarction
