# Correction to: A 20-minute, free-running, whole-heart cardiac MRI to image myocardial injury following reperfused myocardial infarction: demonstration in a large animal model

**DOI:** 10.1093/radadv/umag037

**Published:** 2026-09-08

This is a correction to: Xinheng Zhang, Hsin-Jung Yang, Yuheng Huang, Ghazal Yoosefian, Keyur Vora, Khalid Youssef, Benjamin Wilk, Anthony G Christodoulou, Debiao Li, Behzad Sharif, Frank S Prato, Andreas Kumar, Rohan Dharmakumar, A 20-minute, free-running, whole-heart cardiac MRI to image myocardial injury following reperfused myocardial infarction: demonstration in a large animal model, *Radiology Advances*, Volume 3, Issue 2, March 2026, umag009, https://doi.org/10.1093/radadv/umag009

In the originally published version of this manuscript, author Keyur Vora’s credentials were mistakenly given as ‘PhD’. This should instead be ‘MD, MS’.

This error has been corrected.

