# Supplementary material for: A 20-minute, free-running, whole-heart cardiac MRI to image myocardial injury following reperfused myocardial infarction: demonstration in a large animal model
Source: Radiol Adv. 2026 Apr 17;3(2):umag009. doi: 10.1093/radadv/umag009 (PMC13099398; doi:10.1093/radadv/umag009)
Supplement: umag009_Supplementary_Data [file umag009_supplementary_data.zip › Supplementary_Data.zip!/Supplementary_Material_RadiologyAdvances_revision1_RD_XZ_png_anonymized_v2_Feb9_PDF.pdf]

A 20-minute, Free-Running, Whole-heart Cardiac MRI to Image Irreversible Myocardial Injury Following Reperfused Myocardial Infarction: Demonstration in a Large Animal Model

Xinheng Zhang, PhD[1,2*], Hsin-Jung Yang, PhD[2*], Yuheng Huang, MS[1,3], Ghazal Yoosefian, MS[1], Keyur Vora, PhD[1], Khalid Youssef, PhD[1], Benjamin Wilk, PhD[4], Anthony Christodoulou, PhD[5], Debiao Li, PhD[2,3], Behzad Sharif, PhD[1,6], Frank S Prato, PhD[4], Andreas Kumar, MD, MSc[7], Rohan Dharmakumar[1,6], PhD[†]

[1]Department of Radiology & Imaging Sciences and Krannert Cardiovascular Research Center, Indiana University School of Medicine, Indianapolis, IN, USA

[2]Biomedical Imaging Research Institute, Cedars Sinai Medical Center, Los Angeles, CA, USA

[3]Department of Bioengineering, University of California, Los Angeles, CA, USA

[4] Lawson Health Research Institute, London, ON, Canada

[5]Department of Radiological Sciences, University of California, Los Angeles, CA, USA

[6]Departments of Biomedical Engineering, Purdue University, West Lafayette, IN, USA

[7]Division of Cardiology, Department of Medicine, Northern Ontario School of Medicine, Sudbury, ON, Canada

*Equal Contributions

[†]Correspondence to:

Rohan Dharmakumar, PhD

Cardiovascular Imaging Research Center

Medical Imaging Research Institute

Department of Radiology & Imaging Sciences

Indiana University School of Medicine

E373 – 1800 N Capitol Ave.

Indianapolis, IN, USA 46202-1228

Phone: (317) 274-5652 | Email: rdkumar@iu.edu

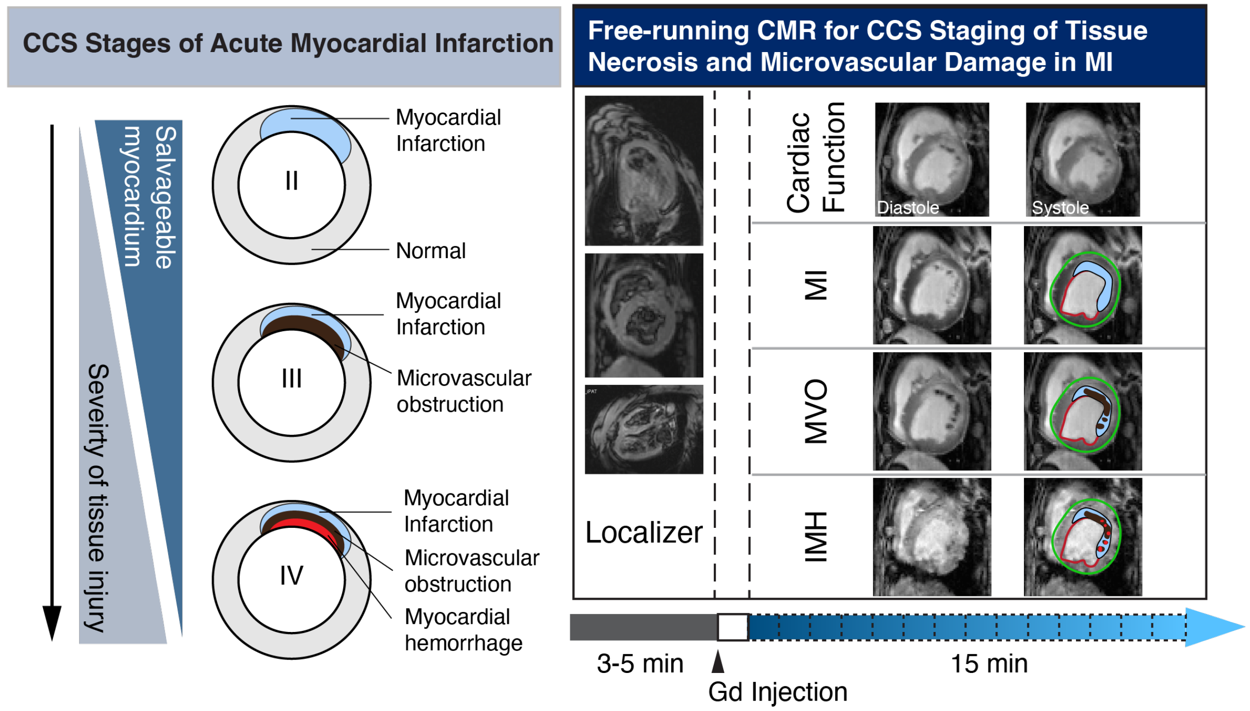

Figure S1. Schematic diagram of Canadian Cardiovascular Society (CCS) staging of irreversible myocardial infarction (Stage II – Stage IV) and proposed free-running CMR protocol within 20 min to determining cardiac function, severity of tissue necrosis and microvascular damage in reperfused MI with whole-heart coverage.

SUPPLEMENTARY MATERIAL 1

METHODS

Sequence diagram

Our low-rank tensor (LRT) approach uses a continuous randomized Gaussian-distributed 3D Cartesian k-space pattern to avoid synchronization between sampling and periodic motion (**Fig. 1A**). The acquisition cycles through a fixed inversion recovery (IR) module, with fast low-angle single-shot (FLASH) excitations for data readout filling the entire recovery period between preparation pulses. Two interleaved data sets are collected during the continuous acquisition: The training auxiliary data ($\boldsymbol{d_{tr}}$) are collected frequently at the k-space center line to provide temporal information, and the imaging data ($\boldsymbol{d_{img}}$) are randomly collected with a 3D Gaussian sampling with Cartesian trajectory to provide spatial information. Each readout consisted of 6 multi-gradient echoes (mGRE), and they are used for both training and imaging data. The runtime for sequence to sample the necessary data is 14 min and 20 sec.

Low-rank tensor imaging model

We employed the LRT framework to model the underlying image as a 6-dimensional image $x(\boldsymbol{r}, T_c, T_r, T_t, T_{t1}, T_{te})$ with one spatial dimension indexed by $\boldsymbol{r} = [x, y, z]$, and five temporal dimensions describing cardiac motion, respiratory motion, temporal dynamics along Gd enhancement, T_1 recovery, and T_2^* decay indexed by $T_c, T_r, T_t, T_{t1}, T_{te}$ respectively. By taking advantage of the spatial-temporal correlations, the image can be factorized as:

$$x(\boldsymbol{r}, T_c, T_r, T_t, T_{t1}, T_{te}) = \sum_{l=1}^{L} u_l(r)\, \phi_l(T_c, T_r, T_t, T_{t1}, T_{te}), \quad (1)$$

$$\phi_l(T_c, T_r, T_t, T_{t1}, T_{te}) = \sum_{j=1}^{J}\sum_{k=1}^{K}\sum_{m=1}^{M}\sum_{n=1}^{N}\sum_{p=1}^{P} g_{ljkmnp}\, c_j(T_c) r_k(T_r) t_m(T_t) w_n(T_{t1}) q_p(T_{te}), \quad (2)$$

where $\{u_l(\boldsymbol{r})\}_{l=1}^{L}$ represent spatial coefficients and $\phi_l(T_c, T_r, T_t, T_{t1}, T_{te})_{l=1}^{L}$ represent temporal functions that can be further decomposed into a core tensor G with elements g_{ljkmnp} and individual bases for each temporal dimension: $\{c_j(T_c)\}_{j=1}^{J}$, $\{r_k(T_r)\}_{k=1}^{K}$, $\{t_m(T_t)\}_{m=1}^{M}$, $\{w_n(T_{t1})\}_{n=1}^{N}$, $\{q_p(T_{t2})\}_{p=1}^{P}$. The combination of Equations 1 and 2 suggests that a discretized image tensor X with elements $x_{ijkmnp} = x(\boldsymbol{r}_i, T_{c,j}, T_{r,k}, T_{t,m}, T_{t1,n}, T_{te,p})$ can be decomposed in Tucker form as follows:

$$X = G \times U_X \times U_C \times U_R \times U_T \times U_{T1} \times U_{TE}, \quad (3)$$

where the columns of U_X, U_C, U_R, U_T, U_{T1}, and U_{TE} contain the basis functions for each corresponding dimension.

The core tensor, and temporal bases are extracted from the training data. We individually decomposed each tensor x_p to reduce computational expenses. Consequently, the subset of the image tensor X at the pth echo can be written as:

$$X_p = \hat{G} \times U_p \times U_C \times U_R \times U_T \times U_{T1}, \quad (4)$$

Where $\hat{G}$ is an updated core tensor without T2* dimension, and U_p are the spatial coefficients for the pth echo. All echoes share the motion and T1 relaxation bases, as changes in motion T1 relaxation bases are negligible on the time scale between the echoes.

Image reconstruction

This work adopts a strategy for image reconstruction with LRT framework from previously published methods[1,2]. Specifically, the 6-way low-rank image tensor was reconstructed with the following steps:

(1) *"real-time" reconstruction of images using explicit low-rank matrix with only one time dimension*: A modified independent component analysis (ICA) algorithm was used to automatically place the corresponding images into 4 respiratory bin and 24 cardiac bins. In this scheme, respiratory motion is identified first, and respiratory bins assigned at each iteration are low-pass-filtered with 40 Hz cutoff frequency. Subsequently cardiac bins are band-pass-filtered with range of 40-130 Hz (a range for possible heart rates). Also, temporal dynamics is evenly divided into 15 sections with the duration of each section of 57 seconds.

(2) *Determining the temporal basis functions of* $\boldsymbol{U}_{T1}$: According to the Bloch equation, a dictionary of feasible IR GRE signal curves is generated using the sequence parameters with a range of T_1 and B_1 inhomogeneity values. The dictionary consists of 21 T_1 values logarithmically spaced from 100 to 3000 ms, 10 flip angles equally spaced between 0.5° to 5°, and 6 inversion efficiency factor equally spaced between -1 (perfect inversion) and -0.5. Basis functions of $\boldsymbol{U}_{T1}$ are extracted from singular value decomposition (SVD) of the dictionary.

(3) *The training tensor* $\boldsymbol{D}_{tr}$: $\boldsymbol{D}_{tr}$ can still be undersampled as training data acquisition cannot typically account for every combination of cardiac phase, respiratory phase, T1 and temporal dynamics dimensions. To address this we applied a small-scale, low-rank tensor completion algorithm by solving the following optimization problem:

$$\widehat{\boldsymbol{D}}_{tr} = \arg \min_{\substack{D_{tr}, s.t. \\ \boldsymbol{D}_{tr,(4)} \in range(\boldsymbol{U}_{T1})}} ||\boldsymbol{d}_{tr} - \Omega_{tr}(\boldsymbol{D}_{tr})||_2^2 + \lambda \sum_{i=1}^{4} ||\boldsymbol{D}_{tr,(i)}||_* + R(\boldsymbol{D}_{tr}), \tag{5}$$

where $\boldsymbol{d}_{tr}$ is the collected training data; $\Omega_{tr}(\cdot)$ is the sampling operator for the training data set; $\boldsymbol{D}_{tr,(i)}$ is the mode-i unfolding of the complete training tensor; $||\cdot||_*$ denotes the matrix nuclear norm and λ weights the nuclear norm penalties and $R(\cdot)$ is a temporal regularizer.

(4) *Extracting temporal basis functions*: Temporal basis function are determined along the cardiac phase, respiratory phase, and T1 recovery dimensions, namely, columns in U_C, U_R, U_T and U_{T1} respectively, as well as the core tensor $\hat{G}$ from the high-order SVD of the completed training tensor $\boldsymbol{D}_{tr}$

(5) *Solve the spatial coefficients* ***U*** *echo-by-echo*:

$$\widehat{U}_p = arg \min_{U_p} \left|\left|\boldsymbol{d}_{img,p} - \Omega\left(\hat{\phi} \times FSU_p\right)\right|\right|_2^2 + R\left(U_p\right) \tag{6}$$

For known temporal factor tensor $\hat{\phi} = \hat{G} \times U_C \times U_R \times U_T \times U_{T1}$, where $\boldsymbol{d}_{img,p}$ are the imaging data at the pth echo, and $R(\cdot)$ is a wavelet sparsity regularizer. The reconstructed image tensor for the pth echo is then given by the product $\hat{X}_p = \hat{\phi} \times \widehat{U}_p$. Each pth echo required 1.5 hours of reconstruction time.

Image Analysis

For cardiac function validation, the endocardium was manually contoured in each slice for both images, and the end-diastole and end-systole phase were identified. Images from late enhancement at segment # 41 was chosen to visualize the optimal contrast between myocardium and blood pool. LVEF was then calculated for each method using the following equation:

$$LVEF = \frac{LVEDV - LVESV}{LVEDV} \times 100\%, \tag{7}$$

where LVEDV and LVESV represents end-diastolic and end-systolic LV volume respectively.

For MI volume and transmurality measurements, for the conventional approach, the IR time was determined from the console to approximate the nulled remote myocardium to maximize the contrast between remote and MI zones. For the proposed approach, nulled remote myocardium was chosen retrospectively from reconstructed tensor for comparison. Similarly, endo- and epi-cardium were drawn and remote myocardial ROI was drawn to identify MI region with Mean+5SD method for each slice from base to apex. MI transmurality was computed with 50-chord analysis and then mean transmurality was calculated by averaging the infarct transmurality across all the chords that have >1% MI extent[3].

MVO measurements were only performed for acute phase acquisitions (MVO is resolved in the chronic phase). End-expiratory, end-diastolic images from the proposed method with nulled remote myocardium, at section 2 and section 15, which represents approximately 2 min and 15 min after contrast injection, were compared with conventional early Gd enhancement (EGE, 2 min) and LGE (15 min). Endo- and epi-cardium were drawn and MVO were identified as hypointense zones within the MI zone thresholded by Mean+5SD method. Volumetric late (persistent) MVO was compared between proposed approach and conventional approach (**Fig. 1B**) using LGE images. For early MVO, single-slices were compared between proposed and conventional approach at different date (Day 6 vs. Day 8).

For IMH volume measurement with the proposed method, late enhancement (section 15) with late T1 recovery (Segment #181, 2376 ms after IR) were used to determine IMH. For conventional methods, IMH was identified on mGRE acquisition before Gd injection with the

same TE = 11.43 ms. For both approaches, endocardium, epicardium and remote ROI were drawn and Mean-2SD method was applied at each slice.

To test the accuracy of regions of interest (MI, MVO and IMH) between two approaches, the slices were first segmented based on AHA 16-segment model. Each segment in conventional 2D cardiac MRI was applied with 1% threshold for Receiver Operating Characteristic (ROC) analysis as a ground truth, while same 16-segment model were applied to slice-matched images using the proposed method as prediction variables.

REFERENCES

1. Christodoulou AG, Shaw JL, Nguyen C, et al. Magnetic resonance multitasking for motion-resolved quantitative cardiovascular imaging. *Nat Biomed Eng*. 2018;2(4):215-226. doi:10.1038/s41551-018-0217-y
2. Cao T, Wang N, Kwan AC, et al. Free-breathing, non-ECG, simultaneous myocardial T1, T2, T2*, and fat-fraction mapping with motion-resolved cardiovascular MR multitasking. *Magn Reson Med*. Published online June 17, 2022. doi:10.1002/mrm.29351
3. Kali A, Cokic I, Tang RLQ, et al. Determination of Location, Size, and Transmurality of Chronic Myocardial Infarction Without Exogenous Contrast Media by Using Cardiac Magnetic Resonance Imaging at 3 T. *Circ Cardiovasc Imaging*. 2014;7(3):471-481. doi:10.1161/CIRCIMAGING.113.001541

SUPPLEMENTARY MATERIAL 2

RESULTS

The measures of LVEF, MI volume, MI transmurality and IMH volume for both acute and chronic subgroups are summarized in **Table S1**.

LVEF at Acute and Chronic Phases of MI

LVEF in acute and chronic phases as determined using the proposed vs. conventional methods were in excellent agreement from Bland-Altman analysis: Acute – mean bias = -0.03%, LoA = -1.9 – 1.8 %, RPC = 1.8 %, Chronic – mean bias = -0.4%, LoA = -2.5 – 1.7 %, RPC = 2.1 %, **Fig. S2B**). Linear regressions showed excellent correlations (**Fig. S2A**, Acute: slope = 0.92, 95% CI: [0.80, 1.03], intercept = 2.56, 95% CI: [-1.14, 6.26], R^2 = 0.97, $P<0.001$, Chronic: slope = 0.99, 95% CI: [0.72, 1.26], intercept = 0.01, 95% CI: [-9.29, 9.32], R^2 = 0.93, $P<0.001$).

Infarct Volume at Acute and Chronic Phases of MI

MI volume in acute and chronic for the proposed vs. conventional were in excellent agreement from Bland-Altman analysis: Acute - mean bias was 0.59%, LoA was -2.7 – 3.9%, RPC was 3.3%, **Fig. S2A**; Chronic - mean bias was 0.50%, LoA was -8.6 – 9.6%, RPC was 9.1%, **Fig. S2C**. MI transmurality in acute and chronic for proposed vs. conventional were in good agreement: Acute - mean bias was -0.32%, LoA was -2.8 – 2.2%, RPC was 2.5%, **Fig. S2B**; Chronic - mean bias was 2.7%, LoA was -4.1 – 9.5%, RPC was 6.8%, **Fig. S2D**. Linear regression of MI volumes between conventional and proposed approaches showed excellent correlation (**Fig. S3A**, slope = 1.07, 95% CI: [0.99, 1.15], intercept = -0.98, 95% CI: [-2.56,

0.60], R^2 = 0.97, P<0.001 for acute phase; **Fig. S3C**, slope = 0.85, 95% CI: [0.48, 1.20], intercept = -1.43, 95% CI: [-2.81, 5.68], R^2 = 0.81, P<0.001 for chronic phase), and for MI transmurality (**Fig. S3B**, slope = 0.92, 95% CI: [0.63, 1.22], intercept = 5.11, 95% CI: [-12.95, 23.16], R^2 = 0.83, P<0.001 for acute phase; **Fig. S3D**, slope = 1.14, 95% CI: [0.72, 1.56], intercept = -4.80, 95% CI: [-27.23, 17.62], R^2 = 0.86, P<0.001 for chronic phase).

Intramyocardial Hemorrhage at Acute and Chronic Phases of MI

IMH volume at acute and chronic phases determined using proposed vs. conventional approaches were in excellent agreement from Bland-Altman analysis: Acute - mean bias was 0.16%, LoA was -2.0 – 1.7%, RPC was 1.8%, **Fig. S4A**; Chronic - mean bias was -0.14%, LoA was -0.8 – 1.1%, RPC was 1.0%, **Fig. S4C**. Linear regression of IMH volume between conventional and proposed approaches showed excellent correlation (**Fig. S4A**, slope = 1.00, 95% CI: [0.71, 1.30], intercept = -0.19, 95% CI: [-2.31, 1.93], R^2 =0.90, P<0.001 for acute phase; **Fig. S4B**, slope = 1.00, 95% CI: [0.78, 1.21], intercept = 0.15, 95% CI: [-0.51, 0.81], R^2 = 0.95, P<0.001 for chronic phase). Significant correlation was found in IMH volume at both acute and chronic phase of MI.

Table S1. Measures of the proposed and conventional approach in cardiac function, myocardial infarction (MI), microvascular obstruction (MVO) and intramyocardial hemorrhage (IMH) characterization divided to acute and chronic sub-groups.

	Acute			Chronic		
	Proposed	Conventional	P-value	Proposed	Conventional	P-value
LVEF (%)	31.72±4.91	34.14±3.81	0.35	30.84±5.66	34.54±3.72	0.32
MI volume (%)	21.58±9.35	21.00±8.57	0.26	11.02±2.74	11.35±2.92	0.47
MI transmurality (%)	60.30±10.95	59.80±10.79	0.71	55.89±8.74	53.19±7.08	0.05
IMH volume (%)	6.60±3.04	6.73±2.88	0.67	2.54±2.16	2.11±4.57	0.41

Abbreviation: LVEF, Left-Ventricle Ejection Fraction; MI, Myocardial Infarction; IMH, Intramyocardial Hemorrhage

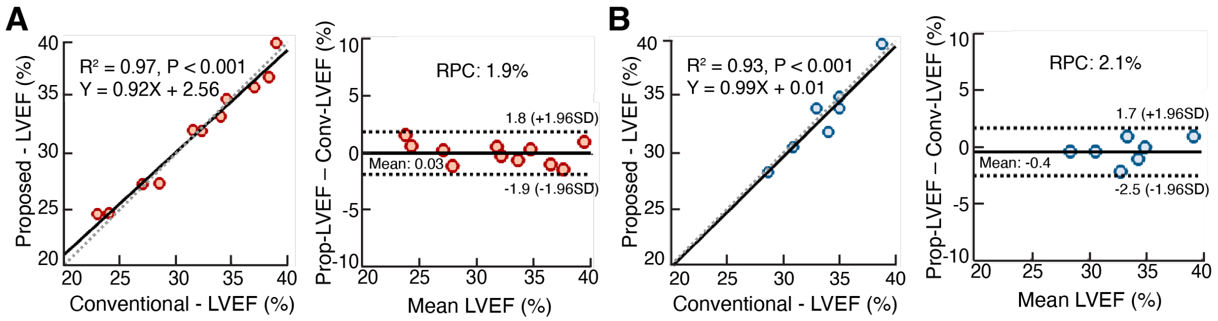

Figure S2. LVEF – Proposed vs Conventional Approaches. **A)** Acute Phase - on left: Findings from linear regression analysis of LVEF between conventional and proposed approaches, fitted equation and R^2 are shown in each plot. On right: Bland-Altman analyses comparing the agreement between the two approaches, reproducibility coefficient (RPC). Only acute data points (red) are shown. **B)** Chronic Phase - Linear regression and Bland-Altman comparing LVEF between two approaches as in A (blue dots).

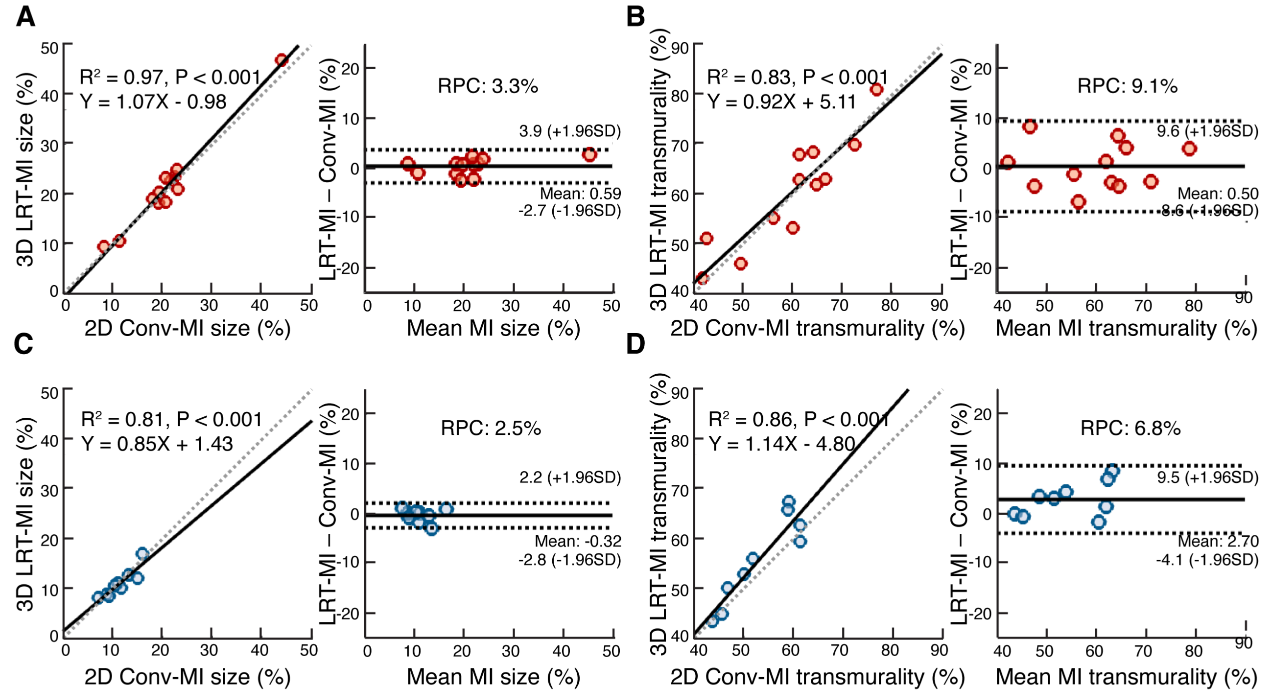

Figure S3. MI Characteristics - Proposed vs Conventional Approaches. **A)** Acute Phase MI Size - On left: Findings from linear regression comparing MI volume between conventional and proposed approaches in the acute phase are shown along with fitted equation and R^2. On right: Bland-Altman analysis comparing agreement between the two approaches are shown along with reproducibility coefficient (RPC) is shown in each plot. **B)** Acue Phase MI Transmurality – Findings from linear regression and bland-Altman analysis of MI transmurality between two approaches with are shown as in A. **C)** & **D)** show findings from linear regression and Bland-Altman analysis of MI volume and MI transmurality respectively in chronic phase.

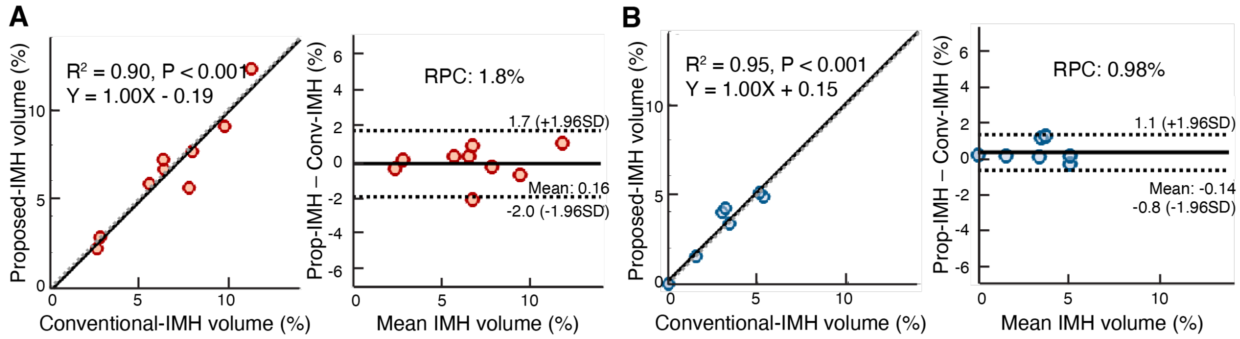

Figure S4. IMH - Proposed vs Conventional Approaches. **A)** Acute Phase IMH - On left: Findings from linear regression of IMH volume between conventional and proposed approaches is shown along with fitted equation and R^2. On right: Findings from Bland-Altman analyses of agreement in IMH volume between the two approaches is shown with reproducibility coefficient (RPC). **B)** Findings from linear regression and Bland-Altman analysis of IMH volume between two approaches are shown as in A but for chronic phase.

SUPPLEMENTARY MATERIAL 3

To assess the edge sharpness/blurriness in the conventional and the proposed methods, we drew a line crossing the edge between the myocardium and blood pool for a line profile. The line profile was then fitted with a sigmoid function. The 10–90% edge rise distance (ERD)[1] was then measured on the image domain to determine the sharpness of each image. The higher ERD value indicates blurrier edge between the myocardium and blood pool. We analyzed all late-enhancement images used for MI characterization in both conventional and proposed approaches.

A representative subject and its line profile was shown for the conventional and proposed in **Fig. S5A** and **Fig. S5B** respectively. The 10-90% ERD is 2.04±1.05 mm for conventional while 10-90% ERD for the proposed method is 3.13±1.27 mm (paired t-test, P < 0.001, **Fig. S5C**). This suggests the proposed methods showed less sharpness between the myocardium and blood pool comparing to the conventional acquisition.

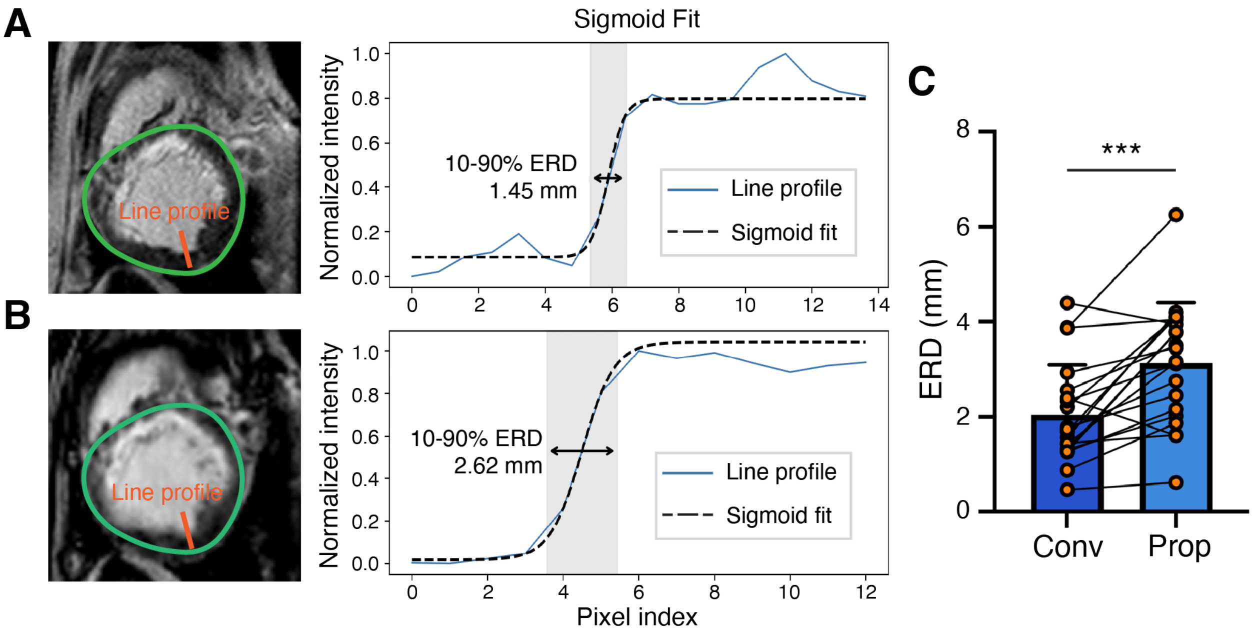

Figure S5. Edge blurriness assessed by line profile between conventional and proposed approaches. **A)** A representative example of a conventional LGE image with line profile put crossing the edge between myocardium and blood pool. Sigmoid fit was applied to the line profile and 10-90% edge rise distance (ERD) was calculated (1.45 mm). **B)** A representative example of a LGE image reconstructed by the proposed framework with line profile on the same subject. Sigmoid fit was applied to the line profile and 10-90% ERD was calculated (2.62 mm). **C)** Bar plot of ERD comparing between the conventional (Conv) and the proposed (Prop) methods, *** denotes P < 0.001.

REFERENCES

1. Fauver M, Seibel E, Rahn JR, Meyer M, Patten F, Neumann T, Nelson A. Three-dimensional imaging of single isolated cell nuclei using optical projection tomography. Opt. Express. 2005; 13:4210–4223.
